# Methicillin-Resistant *Staphylococcus aureus* (MRSA) in Artisanal Cheeses in México

**DOI:** 10.1155/2018/8760357

**Published:** 2018-11-18

**Authors:** Roberto Adame-Gómez, Jeiry Toribio-Jimenez, Amalia Vences-Velazquez, Elvia Rodríguez-Bataz, Maria Cristina Santiago Dionisio, Arturo Ramirez-Peralta

**Affiliations:** ^1^Laboratorio de Investigación en Patometabolismo Microbiano, Universidad Autonoma de Guerrero, Chilpancingo de los Bravo 39000, Mexico; ^2^Laboratorio de Investigación en Microbiologia Molecular y Biotecnologia Ambiental, Universidad Autonoma de Guerrero, Chilpancingo de los Bravo 39000, Mexico; ^3^Laboratorio de Investigación en Inmunobiologia y Diagnostico Molecular, Universidad Autonoma de Guerrero, Chilpancingo de los Bravo 39000, Mexico; ^4^Laboratorio de Investigación en Parasitologia, Universidad Autonoma de Guerrero, Chilpancingo de los Bravo 39000, Mexico; ^5^Laboratorio de Investigación en Analisis Microbiologicos, Universidad Autonoma de Guerrero, Chilpancingo de los Bravo 39000, Mexico

## Abstract

Milk and dairy foods have frequently been implicated in staphylococcal food poisoning, and contaminated raw milk is often involved. The aim of the study was to determine the occurrence of methicillin-resistant *Staphylococcus aureus* (MRSA) in raw cow milk cheese produced in Mexico. A total of 78 unpasteurized cow milk cheese samples were screened for *S. aureus*. The isolates were identified as *S. aureus* based on morphology, Gram stain, catalase test, coagulase test, and mannitol salt agar fermentation. Isolates were subjected to biotyping, the methicillin resistance was analyzed using the disk diffusion, and the Staphylococcus enterotoxin A (SEA) production was examined by a dot-blot analysis. From a total of 78 samples of unpasteurized cheeses analyzed in this study, 44 cheeses were positive for *S. aureus*; however, a differential contamination between the different types of cheeses was observed, with high risk of contamination in adobero cheese (12, 95% CI 1.75 to 94.20; *p*=0.002). In this study, the frequency of methicillin-resistant *Staphylococcus aureus* (MRSA) was 18.1% (8/44) and of enterotoxin A producers was 18.1% (8/44). When classified by biotypes, MRSA only belongs to the human ecovar biotype (2/8, 25%) and the D biotype (4/8, 50%). *S. aureus* producers of enterotoxin A were distributed in specific nonhost biotypes.

## 1. Introduction

Staphylococcal food poisoning (SFP) is one of the most common food-borne diseases worldwide resulting from the consumption of foods containing staphylococcal enterotoxins (SEs) produced mainly by *Staphylococcus aureus* [1]. *S. aureus* is ubiquitous in the environment and can be found in the air, water, humans, and animals [2]. Milk and dairy foods have frequently been implicated in staphylococcal food poisoning, and contaminated raw milk is often involved [3]. The ability of *S. aureus* to grow and produce SEs under a wide range of conditions is evident from the variety of foods implicated in staphylococcal food poisoning [4]. Indeed, milk is a good support for *S. aureus* growth, and dairy products are a known source of intoxication [3]. Furthermore, it has been described that this microorganism is found in human handling, water, milking equipment, and the environment, considered as important sources of contamination [5]. Fresh cheeses are made from whole or low-fat cow's milk curd via casein coagulation with rennet, in many cases, without thermal treatment [6]. A lot cheeses are made in Mexico, including panela, fresco, ranchero, oaxaca, asadero, mozzarella, morral, adobero, and cottage. The fresco is a kind of cheese that is soft, fresh, unpressed, and unripened obtained by raw milk enzymatic coagulation. Requeson is whey cheese, obtained by heating at temperatures between 85 and 90°C, the supernatant is obtained from enzymatic coagulation, and the new coagulated fraction is collected and freely drained. Oaxaca cheese, a fresh pasta “filata” cheese of Mexican origin, is made from raw milk, naturally acidified by the microflora present in milk. Cotija cheese is a Mexican handcrafted product made from raw cow milk whose ripening process occurs spontaneously and, presumably, it is influenced by environmental conditions. Adobero is a kind of cotija cheese, only has differences is in the cheese surface, which is spicy. Many fresh cheese producers in Mexico use unpasteurized (raw) milk because they believe the native microbiota of raw milk confers pleasant aromas and flavors to the final product. A large proportion of cheese consumers also prefer cheeses made with raw milk [7]. However, raw milk products are known to contaminate with *S. aureus*. Even has been described, cheese makers carrying enterotoxin-producing *S. aureus* in their noses or on their hands are thought to be the main source of food contamination caused by physical contact or through respiratory secretions [8]. The aim of the study was to determine the occurrence of methicillin-resistant *Staphylococcus aureus* (MRSA) in raw cow milk cheese. Moreover, enterotoxigenicity of the isolates was investigated.

## 2. Materials and Methods

A total of 78 unpasteurized cow milk cheese samples were collected, between January and March 2016, from street vendors and at local markets from three zones in Chilpancingo, Guerrero, Mexico: San Francisco, Los Angeles, and Caminos. The 78 samples included fresh cheese (15 samples), cotija cheese (15 samples), adobero cheese (16 samples), oaxaca cheese (16 samples), and requeson (16 samples).

All cheese samples were prepared for quantitative analysis of *S. aureus* by homogenizing 25 g cheese and 225 mL 0.8% salt solution for 1 min. Additional 10-fold dilutions were made using sterile 0.8% salt solution. Suitable dilutions were spread plated at volumes of 0.1 mL on Baird–Parker agar supplemented with egg yolk tellurite emulsion (Bioxon) and incubated under aerobic conditions at 37°C. Typical colonies (black, shiny, convex colonies with entire margins and clear zones, with or without an opaque zone) were counted after 24 h of incubation. The detection limits in cheese are 100 CFU/g. If present, 5 egg yolk reaction-positive colonies were chosen from each sample for further identification [9]. All suspect colonies were spread plated on a second culture medium, that is, mannitol salt agar (Bioxon). The isolates were identified as *S. aureus* based on their colony morphology (yellow colonies and a surrounding yellow medium in mannitol salt agar), tellurite reduction, lecithinase activity, and mannitol fermentation and by their ability to coagulate human plasma (tube coagulase test) [10]. Samples were categorized as positive if at least one colony forming unit was isolated. In the samples with more than one colony forming unit, only a colony that fulfilled all the classic characteristics of *S. aureus* (coagulase-positive and mannitol-fermenting staphylococci, tellurite reduction positive) was selected for future characterization. All 44 isolates were biotyped following the method described by Devriese in 1984 [11]. The method includes four phenotypic tests: production of staphylokinase (K), *β*-haemolysin (*β*), coagulation of bovine plasma (BPC), and growth on crystal violet agar (CV) (Table 1). The combination of the test results generate four host-specific biotypes (ecovars), namely, human, poultry, bovine, and ovine and five nonhost-specific biotypes (NHS biotypes) individually named after the test results: K − *β* + CV: C (biotype A), K − *β* + CV: A (biotype B), K + *β* − CV: A (biotype C), K + *β* + CV: A (biotype D), and K − *β* − CV: C (biotype E). The methicillin-resistance testing of the isolates was performed on Mueller–Hinton agar (oxoid) by the disk diffusion method in accordance with Clinical Laboratory Standards Institute Guidelines. The antimicrobial agent tested was cefoxitin (30 *μ*g/disk). *S. aureus* ATCC 25923 was the control strain in every test run [12].

The determination of enterotoxin A was carried out in the laboratory. In brief, the supernatant of 24 h cultures of *S. aureus* (1 × 10^9^ CFU/mL) grown at 37°C in a tube containing 5 mL BHI (brain heart infusion) broth was separated from cell by centrifugation at 8000 × g for 20 minutes. For which sample, 6 *μ*L of supernatant was placed in a nitrocellulose membrane (Santa Cruz Biotechnology, Inc., USA). After blocking in 5% nonfat milk for 1 h, the blots were incubated overnight at 4°C with anti-seA (Abcam, Cambridge, MA, diluted 1 : 5000). Blots were then washed in TBS-T (Tris-buffered saline, 0.1% Tween 20) (20 mM Tris, pH 7.5. 150 mM NaCl 0.1% Tween 20), incubated for 1 h at room temperature with HRP (horseradish peroxidase) conjugated anti-mouse IgG (Abcam, Cambridge, MA, diluted 1:1000), and washed again prior to signal detection with TMB substrate (3,3′,5,5′-tetramethylbenzidine) (Santa Cruz Biotechnology, Inc., USA) for one minute. The dyed spots were observed as purple spots on the membrane, confirming the production of enterotoxin A.

A kind of cheese as risk factors for *S. aureus* contamination was analyzed by Pearson's chi-square test or Fisher's exact test using STATA V.12 for Windows. Variables significant on the univariate model were analyzed by logistic regression to identify independent risk factors. A *p* value <0.05 was considered statistically significant. OR values higher than 1 were considered as risk factors. Each group was compared with the group with less isolates (oaxaca cheese group).

## 3. Results and Discussion

### 3.1. Occurrence of *S. aureus* in Artisanal Cheeses

From a total of 78 samples of unpasteurized cheeses analyzed in this study, 44 cheeses were positive for *S. aureus*; however, a differential contamination between the different types of cheeses was observed, with high risk of contamination in adobero cheese (12, 95% CI 1.75 to 94.20; *p*=0.002), cotija cheese (8.25, 95% CI 1.32 to 56.17; *p*=0.007), and fresco cheese (8.25, 95% CI 1.32 to 56.17; *p*=0.007) (Table 2).

The occurrence of *S. aureus* in this study was 56.4%, which is higher in relation to the studies in Turkey (12.5%) [13] and Iran (16%) [10]; and it is a low frequency in relation to studies in Italy and Serbia, which reports frequencies of 80% [14, 15]. The high frequencies of the bacteria in this study, as well as the others are consistent with a previous study [16], where it is shown that the microorganism can enter the artisanal cheese-making process at different stages of the manual production, with colonized cheese makers representing a likely source of *S. aureus*, as well as studies in Italy [14] and Poland [17], where they described that many strains were present in samples from multiple dairies from different regions and years, highlighting the spread of *S. aureus* in small-scale cheese production plants. The frequency data obtained in this study are important because currently only one study has been carried out for the research of *S. aureus* in Cotija cheese in Mexico in 2014 [18]. Currently, cheese making is one of the most important industries in Mexico, and it uses approximately 25% of the total milk produced in the country and the importance of this industry is reflected in the estimation that around 70% of all Mexican cheeses comes from small-scale productions [19], highlighting the importance of this study. In addition, an outbreak by consumption of artisanal cheese contaminated by *S. aureus* was reported in the area in the year of sampling.

### 3.2. The Distribution of *S. aureus* Biotypes

Various remarkable differences were observed in the distribution of biotypes between cheese isolates. Biotype C was the prevalent biotype (14/44) and present in all the cheeses, and the same way is biotype D. The human ecovar (7/44) was present in cotija, adobero, and fresco cheeses. The frequency of biotypes related to farm is low in this study (poultry ecovar (2/44); bovine ecovar (0/44)) (Table 1).

The ecovar's diversity of the isolated strains makes it possible to explain that the contamination of these cheeses is related to multiple factors during the elaboration process, related from the environment in which the raw material is obtained (porcine ecovar), and to the process and manipulation in a certain stage (human ecovar). In this last point, it has been described that *S. aureus* is a common commensal bacterium of the skin and mucosal membranes of humans [20] and is very vulnerable to destruction by heating and sanitizing agents, so its presence in processed foods is usually an indicative of poor hygiene and/or deficient pasteurization [4].

### 3.3. Occurrence of Methicillin-Resistant *S. aureus* (MRSA) and Enterotoxin A-Producing *S. aureus*

**Figure 1:**
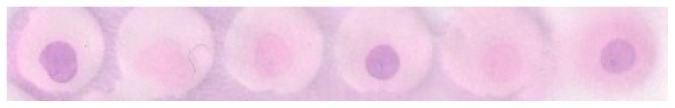
SeA production by *S. aureus* isolated from Mexican artisanal cheeses. From left to right: positive control, negative control, and isolates from cheese.

In this study, the frequency of methicillin-resistant *Staphylococcus aureus* (MRSA) was 18.1% (8/44) and of enterotoxin A producers was 18.1% (8/44) (Figure 1). The enterotoxin A *S. aureus* strains were isolated from all types of cheeses (Table 3).

Of the strains obtained, the prevalence of MRSA was 18.1%; in studies including that in Serbia [15], Italy [14], and Iran [10], no strains were detected with this characteristic; only a study from Brazil with a frequency of 22% [21] which reflects the diversity of strains that circulate in cheeses, emphasizing that only a study monitors MRSA in food [22]. The frequency of enterotoxin A-producing strains was 18.1 %, a fact that is difficult to compare in the sense that a variety of studies have described the enterotoxigenic profile with the search for the genes of interest by PCR [23–25], although the presence of an enterotoxin gene is not a valid indication of SE protein expression [26]. But it is emphasized, even by different techniques, that the enterotoxin chosen in this study is the most frequent among the whole group of enterotoxins [1]. The Mexican legislation establishes *S. aureus* count as an indicator of quality and safety for cheese. In this study, thirty samples showed *S. aureus* above the standard (3.0 log CFU/g) [27] established by Mexican law and isolated in only one of them an enterotoxin A-producing strain (5.6 log CFU/g); the remaining strains were isolated from samples that do not exceed this limit. The permissible limits of *S. aureus* established by the Mexican law are based on that the production of enterotoxins is correlated with proliferation of the bacterium in food, so the bacterial colonies count is used to determine the safety of the product and therefore only consider food samples with a high amount of *S. aureus* to be at risk; however, this number does not ensure the enterotoxigenic capacity of the strain because the influence of the matrix on the produced SE levels has been demonstrated for seD and seiR [28]. In addition, it has been described that preformed enterotoxins can resist heat treatment in products (requiring of 100°C for 5–10 min to be destroyed) [4], where the vegetative cells of *S. aureus* are eliminated; therefore, enumeration by CFU would underestimate this possibility [29]. The same legislation denotes the use of the stable thermonuclease test and indirectly relates it to the production of enterotoxins [30]; therefore, the sensitivity and specificity of this technique was also compared and analyzed with the production of enterotoxin A by dot-blot, which is 60%, considering that the test is not enough to demonstrate the producers of enterotoxins, particularly A. In this sense, there are contradictory studies regarding the power of this test [31, 32], which suggests the search for new rapid indirect or direct tests that allow associating it with enterotoxigenic *S. aureus*. The availability of reliable, rapid, and sensitive SE detection methods is important not only in SFP investigations but also to generate data that allow risk managers to set food safety criteria for SEs and thus prevent SFP.

### 3.4. Relationship between Methicillin-Resistant *S. aureus* (MRSA), Enterotoxin A Producing *S. aureus*, and Biotypes

Fifty percent of the MRSA strains (4/8) correspond to the D ecovar and 25% to the human ecovar (Table 4). At this point, MRSA is spread throughout the world [21] including food handlers that have been described [33, 34], and they are regarded as the main source of food contamination via manual contact or through respiratory secretion [19]. The frequency of enterotoxin A-producing strains is distributed in different ecovars. Studies have shown the relationship between the source of contamination (ecovar) and the type of SE produced by *S. aureus*. SeA and SeB are associated with contamination of human origin [35]. These data can explain the occurrence of enterotoxigenic *S. aureus* in human ecovar and biotype A; however, it has even been described the genes that code for enterotoxins are distributed or present in all origins [36]; this supports the data from the presence of seA in different ecovars of *S. aureus* in this study.

## 4. Conclusions

We conclude that artisanal cheeses in Mexico are contaminated by a large variety of *S. aureus* biotypes with different pathogenic and epidemiological properties. In addition, the risk of contamination is different in each cheese, for which it is necessary to monitor and control the processes involved in the preparation of these foods.

## Figures and Tables

**Table 1:** *Staphylococcus aureus* biotype distribution in Mexican artisanal cheese.

Biotype	*n*	Fresco	Requeson	Cotija	Adobero	Oaxaca
Host-specific ecovars						
Human ecovar	—	—	—	—	—	—
Human ecovar hemolytic	7 (15.9)	4	—	1	2	—
Poultry ecovar	2 (4.5)	1	1	—	—	—
Bovine ecovar	—	—	—	—	—	—
Ovine ecovar	—	—	—	—	—	—

Nonhost-specific (NHS) biotypes						
K − *β* + CV: C^∗^	7 (15.9)	2	2	—	2	1
K − *β* + CV: A^∗^	5 (11.4)	1	1	—	2	1
K + *β* − CV: A^∗^	14 (31.8)	2	2	3	5	2
K + *β* + CV: A^∗^	7 (15.9)	1	1	3	1	1
K − *β* − CV: C^∗^	2 (4.5)	—	—	2	—	—

^∗^Abbreviations are derived from staphylokinase (K), *β*-haemolysin (*β*), and crystal violet growth type (CV). ^∗^^∗^NHS biotypes in this study correspond to the following: K − *β* + CV: C (biotype A), K − *β* + CV: A (biotype *β*), K + *β* − CV: A (biotype C), K + *β* + CV: A (biotype D), and K − *β* − CV: C (biotype E).

**Table 2:** Differential contamination by *S. aureus* in Mexican artisanal cheese.

Variable	Contamination status			Odds ratio^a^		Log CFU/g^b^
	Positive	Negative	OR	95% CI	*p* value	
Cheese						
Fresco	11	4	8.25	(1.32–56.17)	0.007	5.65 ± 1.55
Requeson	6	10	1.18	(0.31–11.14)	0.445	4.31 ± 1.27
Cotija	11	4	8.25	(1.32–56.17)	0.007	5.05 ± 1.39
Adobero	12	4	12	(1.75–94.20)	0.002	5.34 ± 1.43
Oaxaca	4	12		1		4.54 ± 2.08

^a^Calculated by logistic regression model; ^b^the results represent median ± standard deviation.

**Table 3:** MRSA and *S. aureus* enterotoxigenic in artisanal Mexican cheese.

Cheese	MRSA, *n* (%), *N* = 8	SeA, *n* (%), *N* = 8
Fresco	3 (37.5)	2 (25)
Requeson	3 (37.5)	1 (12.5)
Cotija	1 (12.5)	1 (12.5)
Adobero	1 (12.5)	2 (25)
Oaxaca	—	2 (25)

MRSA: methicillin-resistant *Staphylococcus aureus*; seA: enterotoxin A-producing *Staphylococcus aureus*.

**Table 4:** Methicillin resistance and enterotoxin A production in *S. aureus* biotypes.

Biotype	MRSA, *n* (%), *N* = 8	seA, *n* (%), *N* = 8
Host-specific ecovars		
Human ecovar	—	—
Human ecovar *β*+^∗^	2 (25)	—
Poultry ecovar	1 (12.5)	1 (12.5)
Bovine ecovar	—	—
Ovine ecovar	—	—

Nonhost-specific (NHS) biotypes		
K − *β* + CV: C^∗^	—	1 (12.5)
K − *β* + CV: A^∗^	—	2 (25)
K + *β* − CV: A^∗^	1 (12.5)	4 (50)
K + *β* + CV: A^∗^	4 (50)	—
K − *β* − CV: C^∗^	—	—

^∗^Abbreviations are derived from staphylokinase (K), *β*-haemolysin (*β*), and crystal violet (CV) growth type. NHS biotypes in this study correspond to the following: K − *β* + CV: C (biotype A), K − *β* + CV: A (biotype *β*), K + *β* − CV: A (biotype C), K + *β* + CV: A (biotype D), and K − *β* − CV: C (biotype E). MRSA, methicillin-resistant *Staphylococcus aureus*. SeA, enterotoxin A-producing *Staphylococcus aureus*.

## Data Availability

The data used to support the findings of this study are available from the corresponding author upon request.
